# Dynamic cellular maps of molecular species: Application to drug-target interactions

**DOI:** 10.1038/s41598-018-19694-3

**Published:** 2018-01-18

**Authors:** Carolina García, Alejandro Losada, Miguel A. Sacristán, Juan Fernando Martínez-Leal, Carlos M. Galmarini, M. Pilar Lillo

**Affiliations:** 1 Departamento de Química Física Biológica, Instituto de Química-Física “Rocasolano” (CSIC), Madrid, Spain; 2 Departamento de Biología Celular y Farmacogenómica, Pharma Mar S.A., Colmenar Viejo, Madrid, Spain

## Abstract

The design of living cell studies aimed at deciphering the mechanism of action of drugs targeting proteins with multiple functions, expressed in a wide range of concentrations and cellular locations, is a real challenge. We recently showed that the antitumor drug plitidepsin (APL) localizes sufficiently close to the elongation factor eEF1A2 so as to suggest the formation of drug-protein complexes in living cells. Here we present an extension of our previous micro-spectroscopy study, that combines Generalized Polarization (*GP*) images, with the phasor approach and fluorescence lifetime imaging microscopy (*FLIM*), using a 7-aminocoumarin drug analog (APL^*^) as fluorescence tracer. Using the proposed methodology, we were able to follow in real time the formation and relative distribution of two sets of APL-target complexes in live cells, revealing two distinct patterns of behavior for HeLa-wt and APL resistant HeLa-APL-R cells. The information obtained may complement and facilitate the design of new experiments and the global interpretation of the results obtained with other biochemical and cell biology methods, as well as possibly opening new avenues of study to decipher the mechanism of action of new drugs.

## Introduction

The two isoforms of the alpha subunit of eukaryotic translation elongation factor, eEF1A1 and eEF1A2, are highly abundant and multifunctional proteins. Their cellular expression in mammals varies depending on the tissue. While eEF1A1 is present almost ubiquitously, eEF1A2 expression has been shown to be restricted to brain, muscles, heart, islet cells in the pancreas and endocrine cells in the gut of healthy individuals^1^. Moreover, the isoform eEF1A2 is overexpressed in many human tumors and transformed cell lines, favoring tumor cell proliferation while inhibiting apoptosis^1,2^. Plitidepsin (Aplidin; APL) is a cyclic depsipeptide originally isolated from the marine tunicate *Aplidium albicans*. It showed potent anticancer activity in preclinical assays as well as in several phase I/II clinical studies in humans^3^. APL has recently completed a pivotal phase III clinical trial for multiple myeloma (clinical trials. gov identifier: NCT01102426). Interestingly, ectopic expression of eEF1A2 in APL resistant, HeLa-APL-R cells, restores sensitivity to APL. For a current review on the mechanism of action of plitidepsin see ref. ^3^.

Previous *in vitro* and living cell studies strongly suggested that eEF1A2 was the primary target of plitidepsin^4^. We showed that APL interacts with eEF1A2, purified from rabbit muscle, with a *K*_*D*_ of 80 nM. In addition, using a coumarinated analog of APL as FRET donor (APL^*^), and eEF1A-GFP as FRET acceptor overexpressed in HeLa-wt and HeLa-APL-R cells, and FLIM-FRET-phasor analysis in living cells, we detected [APL^*^/eEF1A2-GFP] FRET complexes distributed all over the cell, including the plasma membrane, and/or regions close to the inner face of the membrane, maintaining approximately the same cellular distribution as the elongation factor presented before the treatment of cells with APL. The apparent concentration of APL^*^ FRET complexes, localized at the membrane and/or cell cortex regions, was much lower than areas of the cellular interior, remaining nearly constant over time regardless of the total concentration of APL added to the cells.

A number of important questions arise from this initial study; namely: (*a*) are all the APL-target complexes of the same type? (*b*) does APL have a different microenvironment for each of them? (*c*) how many APL-target complexes are we able to distinguish in a plitidepsin-treated cell? (*d*) are they accumulating in specific cellular regions or evenly distributed all over the cell? (*f* ) how does the population of APL-target complexes change over time? (*g*) which molecular species are key elements for the mechanism of action of the drug? (*h*) are there differences between sensitive HeLa-wt and APL-resistant HeLa-APL-R cells in terms of APL molecular species?

To address these questions, in this study, we have used the 7-dimethylamino coumarin (DMAC^*^) plitidepsin analog, APL^*^ (FRET donor^4^), as a fluorescent tracer to discriminate between APL-target complexes. Generally, the spectroscopic parameters of 7-aminocoumarins derivatives show high sensitivity to changes in microenvironment polarity, polarizability, viscosity/confinement, as well as on the solute-solvent interaction or some interactions with the surrounding media^5–9^. These fluorophores show large Stokes shifted fluorescence emission in polar environments, along with changes in their molar absorption coefficient, fluorescence quantum yield, and excited-state lifetimes. APL^*^ presents the fluorophore DMAC^*^ located on the side-chain of the depsipeptide. Since APL^*^ maintains its bioactivity, the presence of DMAC^*^ should not modify the conformation of the active macrocycle, but the spectroscopic properties of DMAC^*^ may be sensitive to the composition of the APL^*^-target complexes, and/or the characteristics of the cavity/microenvironment in its binding site. DMAC^*^ have at least two hydrogen acceptor sites: the carbonyl oxygen connected to the pyrone ring (strong hydrogen bond with alcohols and water molecules), and the amino-nitrogen. Additionally, in highly polar solvent, the excited state of DMAC^*^ exists both in twisted intramolecular charge transfer TICT, and intramolecular charge transfer ICT. Relaxation towards an ICT state may be accompanied by internal rotation of the dimethylamino group about single bonds within the fluorophore, until it is twisted at the right angle and the conjugation is lost (full charge transfer/stabilized by the polar solvent molecules). No rotation leads only to partial charge transfer. Moreover, hydrogen bond donor groups competing with water molecules, different polarities, and/or microenvironments that facilitate/hinder internal rotation about single bonds of the dimethylamino group in DMAC^*^, may give rise to distinct spectroscopic properties for each possible APL^*^ molecular species, which are part of their characteristic spectroscopic fingerprint.

In this study, we have monitored the formation/inhibition, location, and accumulation of two types of drug-target complexes in real time, in APL sensitive and resistant cells, from two-color fluorescence intensity and lifetime FLIM images of APL^*^ treated cells, acquired simultaneously. We extended the use of the Generalized Polarization approach *GP*^10^ to graph, quite efficiently, at the subcellular level, the relative distribution of two sets of drug-target complexes in living cells, APL^*^-A and APL^*^-B. This simple representation shows, in a qualitative way but with a high degree of contrast, which cellular regions are enriched in each molecular species, and the effects of treatment duration and total concentration of the added bioactive compound, APL. The corresponding fluorescence lifetime FLIM images, analyzed with the phasor approach, has allowed us to quantify fractional intensity contributions of APL^*^-A, APL^*^-B complexes, after substracting the cell autofluorescence AF, pixel by pixel, in time slots of few minutes at sub-μm resolution.

We have found that the formation of APL*-B complexes is highly inhibited in HeLa-APL-R, hence APL*-B complexes seem to be key elements for the mechanism of action of the drug.

Further work has to be done to characterize all the proteins involved in both sets of APL^*^-complexes, and to measure their absolute concentrations over time, in all the different cellular locations, taking into account changes in their fluorescence quantum yields and the cell autofluorescence fractional intensity (mainly from NADH), not negligible for regions showing low concentration of APL^*^-complexes. It is important to highlight here the clever approach presented in a recent work by Ma *et al*.^11^ to measure absolute concentrations of free and bound NADH in cells, using the FLIM-phasor method, a calibration sample of known concentration, and unmodulated light added to FLIM images. Among other micro-spectroscopy approaches using microenvironment sensitive spectral properties of fluorophores to characterize cellular processes in living cells, mention should be made of membrane studies using Laurdan^12–14^, di-4-ANEPPDHQ^15,16^, and NBD^17^ fluorophores, using Generalized Polarization, *GP*, and the FLIM-phasor approaches.

The methodology used in this work is of general applicability to characterize the kinetics of formation/inhibition of drug-target complexes in living cells, whenever it is possible to label them with specific fluorescent probes that are sensitive to the drug microenvironment.

## Results

### Discrimination and subcellular relative distribution of APL^*^ molecular species in living cells

#### Effect of the total concentration of added APL and treatment time from steady-state fluorescence intensity and GP images

We have used the 7-dimethylamino coumarin (DMAC^*^) plitidepsin analog, APL^*^ (FRET donor^4^), as fluorescent tracer, and the simultaneous detection of a set of fluorescence properties, pixel by pixel in the blue (channel *#*1, CH1) and green (channel *#2*, CH2) emission regions, to separate APL^*^-target complexes in two groups, APL^*^-A, with DMAC^*^ in a less polar environment, and APL^*^-B, with DMAC^*^ in a more polar environment. Note that the apparent pixel resolution in the measured *XY* cell images is 200 × 200 nm, but the effective two-photon detection volume would correspond to a Gaussian-Lorentzian beam profile^18^ to about 350 nm laser beam waist and 500 nm axial width, focused at the surface of the coverslip (*Z* = 0), characterized from calibrated samples (data not shown). It is important to note that we do not have enough resolution at the cellular edges to discriminate between the inner or outer leaflet of the plasma membrane, or the region next to the inner leaflet (cell cortex). All these locations will be referred to here as the “membrane” region. Moreover, fluorescence signals measured at the “cytoplasm” region will contain also some contribution from the cellular adhesion area to the coverslip.

We have carried out a thorough analysis of the effect of the total concentration of added drug, [APL], ranging from 2 nM to 450 nM, on APL-target cellular interactions, in living HeLa-wt and HeLa-APL-R cells. For each cell type, at least 20 to 30 cells have been analyzed. For each [APL^*^], we have monitored APL^*^ cell labelling every few minutes from the first minute of interaction to over one hour. Supplementary Fig. S2a–d show a comparative view of two-photon steady-state fluorescence intensity *XY* sections (*Z* = 0) at CH1 and CH2 of representative groups of HeLa-wt and HeLa-APL-R cells, treated with [APL^*^] 5 nM, 10 nM, and mixtures [APL^*^]/[APL], 10 nM/90 nM and 10 nM/440 nM, after ∼20 minutes. Supplementary Fig. S2i,j show the enlarged images of the same group of HeLa-wt cells treated with APL^*^ 10 nM for 20 minutes (*XY* sections) and 60 minutes (*XZ* sections). *XZ* sections indicate that APL^*^ species accumulated preferentially near the surface of the coverslip. Pattern of APL^*^ cellular labelling was totally different for sensitive and APL resistant cells. In HeLa-wt, CH1 intensity images show a broad labeling of the “cytoplasm”, with an average fluorescence intensity signal increasing over time, until an intensity plateau is reached, while CH2 images show characteristic accumulations at specific regions from the first minute of interaction. Comparing the average fluorescence intensities, from both channels, we can conclude that APL^*^-B species (detected mainly from CH1) are the majority species in the “cytoplasm” in HeLa-wt cells for [APL^*^] < 100 nM. No significant changes in intensity were observed when washing with Tyrode-glucose buffer, or adding extra unlabeled APL. Nevertheless, at higher concentrations (100–450 nM) and longer times, the formation of APL^*^-A species in the “cytoplasm” region apparently increases at a higher rate than APL^*^-B species, with very significant changes in the organization of APL^*^-A species, when washing cells with Tyrode-glucose buffer. Furthermore, the most noteworthy result is that labelling of the “cytoplasm” of HeLa-APL-R cells, as detected at CH1, is much smaller than that observed for HeLa-wt cells, for the same added [APL^*^] and treatment time conditions, while the CH2 signal is very similar. In HeLa-wt and HeLa-APL-R cells, the “membrane” region was labelled with APL^*^ from short times (*t* < 5 minutes), both in CH1, and CH2 images, showing fluorescence intensity signals significantly lower than in the “cytoplasm” region, and the values did not change over interaction time, regardless added [APL^*^].

**Figure 1:**
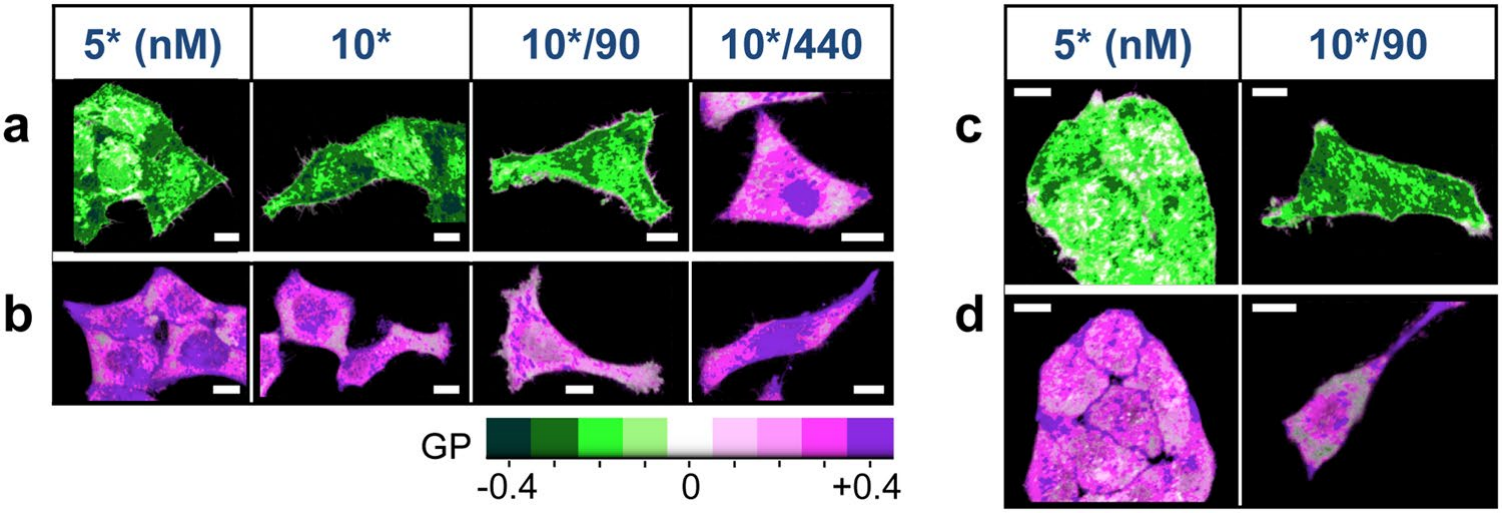
Subcellular relative distribution of APL^*^ species and effect of EGCG 100 μM pre-treatment from *GP* images. Generalized Polarization *GP* images of *XY* sections (at the surface of the coverslip; *Z* = 0) of representative groups of (**a**) HeLa-wt, (**b**) HeLa-APL-R cells, and (**c**) HeLa-wt and (**d**) HeLa-APL-R cells, pre-incubated with EGCG 100 μM for 30 minutes, all treated with different concentrations of added [APL^*^] 5-10 nM, and [APL^*^]/[APL] 10 nM/90 nM, and 10 nM/440 nM for *t* ∼ 20 minutes. Scale bar 10 μm. Dark green-white-blue violet color *GP* scale: Dark green and blue violet colors highlight cellular regions enriched in APL^*^-B (polar) and APL^*^-A (less polar) complexes, respectively. λ_exc_ = 750 nm. CH1: FF01 520/35; CH2: FF02 435/40, Dichroic filter: FF458-Di02. 1.2 ms/pixel.

Figure 1a,b show the *GP* images, calculated from CH1 and CH2 fluorescence intensity images presented in Supplementary Fig. S2, using a Green-Magenta color scale. Dark green, and blue violet colors correspond to cellular regions enriched in APL^*^-B (polar) and APL^*^-A (less polar) species, respectively. White color pixels reveal regions in which the ratio of the total fluorescence intensities (*I*_*F*1_*/I*_*F*2_), measured at CH1 and CH2, is 1.0. In general, in HeLa-wt cells we have observed a high cell to cell variability in terms of the kinetics of internalization of APL^*^. Interestingly, the cells at the edges of the group were labelled more rapidly than interior cells. However, for added [APL^*^] < 100 nM, and *t* ∼ 20–60 minutes, all cells reach a common *GP* distribution pattern at the “cytoplasm” with average *GP* values from −0.4 to −0.1 (average ratio *I*_*F*1_*/I*_*F*2_ ∼ 2.3–1.2). At the “membrane” region, we found a gradient of *GP* values, from +0.4 to 0.0, corresponding to average ratios *I*_*F1*_*/I*_*F*2_ ∼ 0.4–1.0, indicating the presence of APL^*^-A and APL^*^-B populations is more balanced. Furthermore, *GP* images of HeLa-wt cells treated with 2 nM [APL^*^] revealed interesting details of the first steps on the entry path of APL into the cell (Supplementary Fig. S3c). At low [APL^*^], short time interaction *GP* images show average *GP* values from +0.15 to +0.25 (*I*_*F*1_*/I*_*F*2_ ∼ 0.7–0.6) in most of the “cytoplasm” regions, but close to the cell edges the average *GP* values range from −0.15 to −0.25 (*I*_*F1*_*/I*_*F2*_ ∼ 1.4–1.7). This result may indicate that APL^*^-B complexes are first formed on, or near the plasma membrane, and then, somehow overtime, they are distributed throughout the cytoplasm, while APL^*^-A complexes are distributed in the “cytoplasm” in the first minutes of APL^*^ interaction. At longer treatment times (*t* ∼ 90 minutes), *GP* images (data not shown) of 2 nM [APL^*^] treated cells were similar to those of 5–10 nM [APL^*^] treated cells at *t* > 20–30 minutes. Treatment of cells with higher [APL] resulted in a completely different *GP* pattern overtime. *GP* images from HeLa-wt cells treated with [APL^*^]/[APL], 10 nM/90 nM, *t* > 30 minutes, and 10 nM/440 nM, *t* > 10 minutes (Fig. 1a and Supplementary Fig. S3d) showed average *GP* values from +0.25 to +0.5 (*I*_*F1*_*/I*_*F2*_ ∼ 0.6–0.3), indicative of a higher proportion of APL^*^-A complexes practically all over the “cytoplasm”, with accumulations mainly near the cellular edges and the nucleus regions (magenta/blue violet colors in *GP* images).

*GP* images of HeLa-APL-R cells treated with [APL^*^] looked very similar to that of HeLa-wt cells treated with [APL] > 10 nM and long treatment times (Fig. 1b), indicating similar intensities ratios *I*_*F1*_*/I*_*F2*_ ∼ 0.6–0.3.

#### Effect of epigallocatechin-gallate (EGCG) 100 μM pre-treatment

EGCG is an anti-oxidant from green tea that has been shown to affect the structure of lipid rafts. Pre-treatment of HeLa-wt and HeLa-APL-R cells with EGCG 100 μM for 30 minutes resulted in a slowdown of the kinetics of internalization of APL*, especially as detected by CH1 (Supplementary Fig. S2e–h). At equal added [APL*] and similar treatment times, *t*, a slight reduction of averaged *GP* at the “cytoplasm” was observed with respect to cells not treated with EGCG, together with an apparent redistribution of APL^*^ species showing accumulations of APL^*^-A species close to the edges of the cells (magenta/blue violet colors in *GP* images, Fig. 1c,d). This redistribution appeared much more marked in the case of HeLa-APL-R cells.

APL^*^ labelling pattern showed similar characteristics for all analyzed cells of the same type. Supplementary Fig. S5 shows in a single comparative panel a representative sample of *GP* images of groups of analyzed HeLa-wt and HeLa-APL-R cells, treated with APL.

### Fractional contribution of APL^*^ species to the total fluorescence intensity determined pixel by pixel: FLIM-phasor analysis

*GP* imaging approach is simple and very informative. It provides a fast comparison of the effects of added [APL], drug activators/inhibitors on the kinetic of complex formation, but it falls short when quantifying populations of APL^*^ molecular species, especially when the bleedthrough of the other species in each emission channel and the autofluorescence AF contribution is not negligible. We have used the phasor approach to analyze FLIM images using a three species system: two types of APL^*^ complexes, APL^*^-A and APL^*^-B, and the cell autofluorescence, AF, with different lifetimes in the two emission regions AF_1_ and AF_2_ (see Methods). Phasors from cellular regions containing nearly pure APL^*^-A species (detected from CH2) lie on the universal circle, indicating that DMAC^*^ in these species presents a single lifetime, *τ*_*A*_ = 3.9 ns. Analogously, phasors from nearly pure APL^*^-B species (detected from CH1) present a single lifetime, *τ*_*B*_ = 2.45 ns.

#### HeLa-wt cells ± EGCG

**Figure 2:**
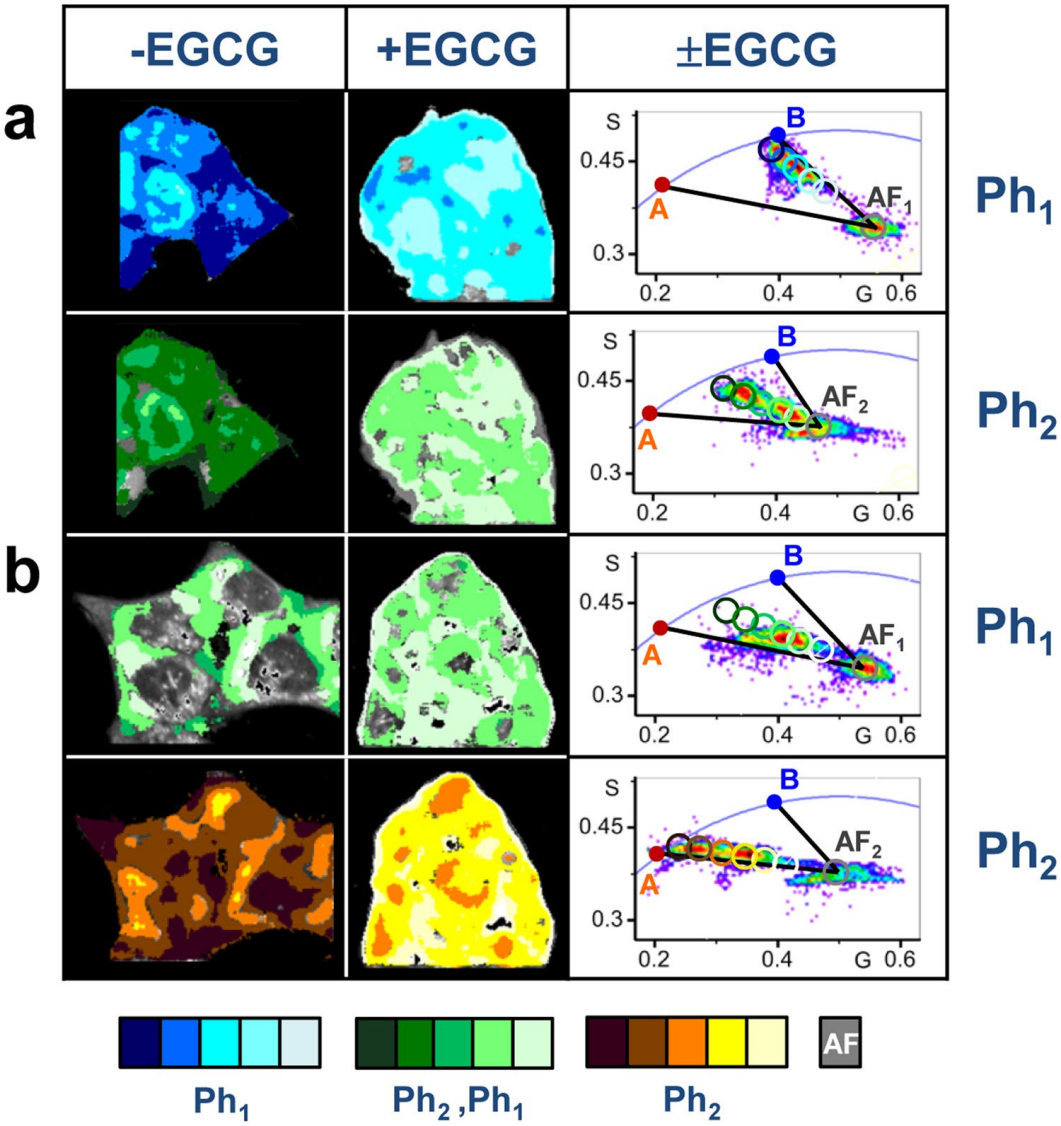
Subcellular relative distribution of APL^*^-target species and effect of EGCG 100 μM pre-treatment from Phasor analysis of the FLIM images. Phasor color maps of FLIM *XY* sections, determined at CH1 (*Ph*_*1*_) and CH2 (*Ph*_*2*_), no treated (-EGCG; first column), and pre-incubated with EGCG 100 μM for 30 minutes (+EGCG; second column) previous to [APL^*^] addition, of representative groups of (**a**) HeLa-wt, and (**b**) HeLa-APL-R cells, treated with [APL^*^] 5 nM for *t* ∼ 20 minutes, superimposed on a general grayscale intensity image of the whole group of cells. Phasor Plots (third column) showing the changes in the phasor cluster location associated with the formation of the different APL^*^-target species at different times, identified in the phasor plot with the corresponding color cursors. Cellular regions showing the maximum fractional contribution of APL^*^-B complexes at CH1 (*Ph*_*1*_; blue scale cursors); cellular regions in which coexist with similar fractional intensities APL^*^-A and APL^*^-B complexes at CH2 (*Ph*_*2*_; green scale cursors); and cellular regions showing the maximum fractional contribution of APL^*^-A complexes at CH2 (*Ph*_*2*_; brown scale cursors). Light to dark colors indicate low to high total APL^*^ fluorescence intensity pixels, high to low fractional contribution of the AF to the total fluorescence intensity, with similar fractional contributions to the total pixel intensity. Black lines in the phasor plots represent the linear combination of 100% pure APL^*^-A complexes and autofluorescence AF (A-AF_1_ at CH1, and A-AF_2_ at CH2), and 100% pure APL^*^-B complexes and autofluorescence AF (B-AF_1_ at CH1, and B-AF_2_ at CH2). λ_exc_ = 750 nm. Channel *#1*: FF01 520/35, Channel *#2*: FF02 435/40, Dichroic filter: FF458-Di02. 1.2 ms/pixel.

Figure 2a shows the phasor color maps and the phasor plots of representative groups of HeLa- wt cells treated with [APL^*^] = 5 nM, *t* = 20 minutes, measured simultaneously at emission channels CH1 (first row) and CH2 (second row). Most of the phasors from the “cytoplasm” FLIM images at CH1 were clustered in locations close to the two-species black line (B-AF_1_). High APL^*^ labelled areas correspond to phasors close the 100% pure APL^*^-B phasor, while low labelled areas approach AF phasor (Fig. 2a third column). A series of blue cursors have been placed in a gradient of color intensity. Light to dark colors correspond to low to high total APL^*^ fluorescence intensity pixels at CH1, high to low fractional contribution of the AF to the total fluorescence intensity, with similar fractional contributions to the total pixel intensity. Note that the dark blue regions in the phasor map images (Fig. 2a first column) coincide with the dark green regions in the *GP* images of the same group of HeLa-wt cells. This is an important result because these two types of images have been obtained by independent methods, and they are providing similar information. On the other hand, most of the phasors from the “cytoplasm” FLIM images at CH2 were clustered in locations between the two black lines (A-AF_2_) and (B-AF_2_) (Fig. 2a third column). Phasors from high APL^*^ labelled areas approach the midpoint of the two-species line (A-B), corresponding to fractional contributions to the total intensity, *α*_*A*_ ≈ *α*_*B*_ ≈ 0.5, *α*_*AF*_ ≈ 0, indicating that APL^*^-A and APL^*^-B species contribute roughly equally to the total fluorescence signal from the “cytoplasm” region, detected at CH2.

Phasor analysis of HeLa-wt cells treated with 2 nM APL^*^ (Supplementary Fig. S3a,b) provides an interesting result. The population of APL^*^-A species remains practically constant for up to 45 minutes (yellow color in the phasor color map), while the population of APL^*^-B species increases slowly (light to dark green color).

Pre-treatment of HeLa-wt cells with 100 μM EGCG for 30 minutes resulted in an important decrease of the population of APL^*^-A and APL^*^-B species at the “cytoplasm”, comparing FLIM images at similar concentration and treatment times, without significant changes with respect to HeLa-wt cells not treated with EGCG (Fig. 2a second column).

#### HeLa-APL-R cells ± EGCG

Figure 2b shows the phasor color maps and the phasor plots of a representative group of HeLa- APL-R cells treated with [APL^*^] 5 nM *t* = 20 minutes, measured simultaneously at emission channels CH1 (first row) and CH2 (second row). Most of the phasors from FLIM images at CH1 were clustered in locations between the two black lines (A-AF_1_) and (B-AF_1_) (Fig. 2b third column), and nearly approach the midpoint of the two-species line (A-B). This result is compatible with the corresponding fluorescence intensity and *GP* images for HeLa-APL-R cells, indicating that APL^*^-A and APL^*^-B species contribute roughly equally to the total fluorescence signal from the “cytoplasm” region, detected at CH1. Note that the “membrane”-region was not colored in the phasor image *Ph*_*1*_ (gray scale overlay fluorescence intensity image), because the corresponding phasors were located closer to the black line (A-AF_1_), indicating that in these regions APL^*^-A species were the majority. On the other hand, most of the phasors from FLIM images at CH2 were clustered quite close to the two-species black line (A-AF_2_) (Fig. 2b third column). High APL^*^ labelled areas correspond to phasors close the 100% pure APL^*^-A phasor, while low labelled areas approach AF phasor. Interestingly, pre-treatment of HeLa-APL-R cells with EGCG 100 μM for 30 minutes resulted in a significant decrease of the population of APL^*^-A and APL^*^-B molecular species in the “cytoplasm”, without significant changes with respect to HeLa-APL-R cells not treated with EGCG (Fig. 2a second column). This result is very similar to that obtained for EGCG treated HeLa-wt cells.

**Figure 3:**
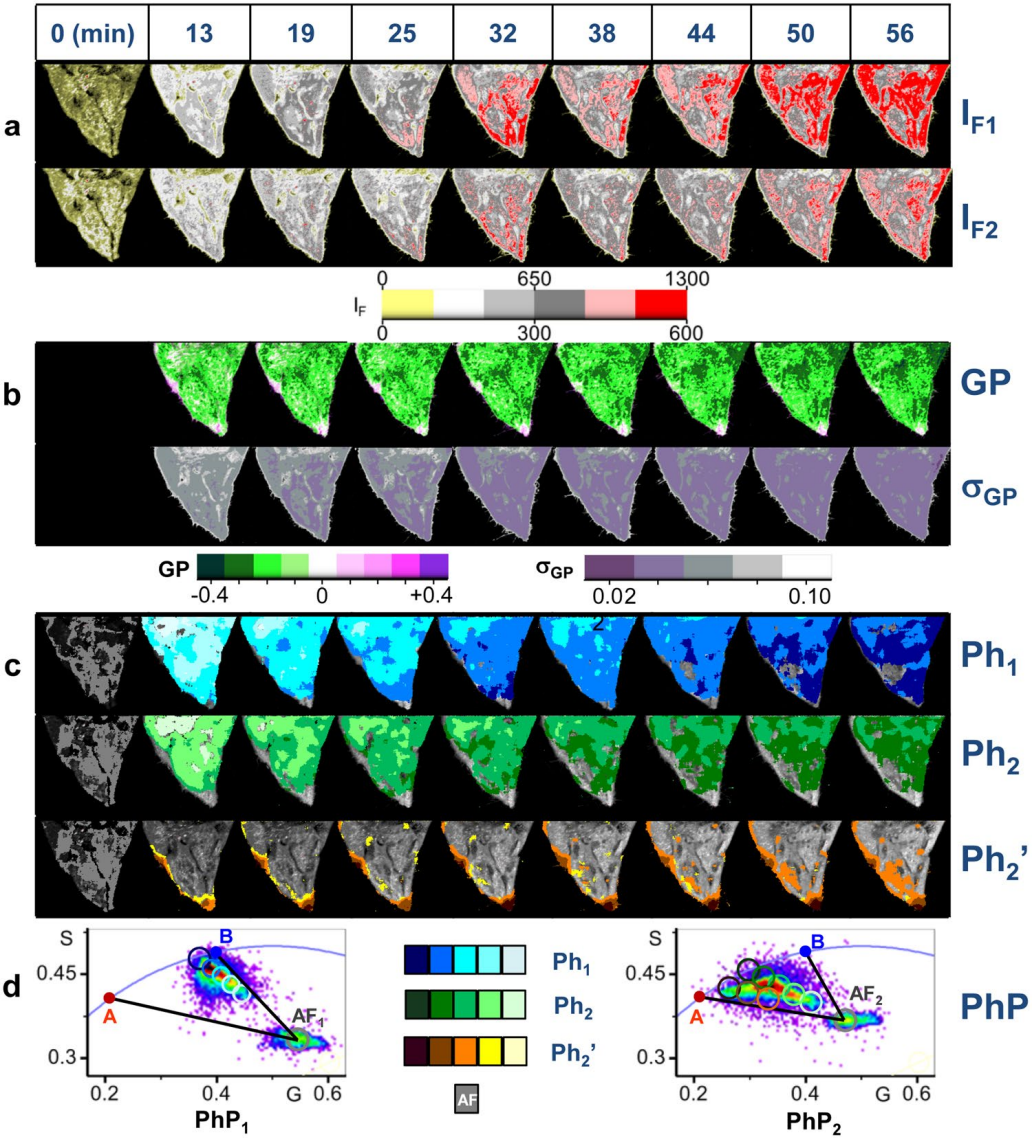
Summary of the whole set of spectroscopic images acquired in this work for each APL treatment condition. (**a**) Two-photon steady-state fluorescence intensity *XY* images of a representative group of HeLa-wt cells treated with [APL^*^] 5 nM. False color-code intensity scale yellow-red. CH1: FF01 520/35 (maximum intensity scale *IF*: 1300 counts). CH2: FF02 435/40 (*IF*: 600 counts). Dichroic filter: FF458-Di02. (**b**) Generalized Polarization *GP* and corresponding standard error *σ*_*GP*_ images. Dark green-white-blue violet color *GP* scale: [−0.45, +0.45] and 5 color scale for *σ*_*GP*_: [0.01, 0.11]. (**c**) Phasor color maps of FLIM *XY* sections, determined at CH1 (*Ph*_*1*_) and CH2 (*Ph*_*2*_). First row (blue scale cursors): regions with the highest APL^*^-B fractional contributions at CH1. Second row (green scale cursors): regions in which APL^*^-B and APL^*^-A species coexist with similar fractional intensity contributions at CH2. Third row (brown cursor scale): highest fractional contribution regions for APL^*^-A at CH2, superimposed on a general grayscale intensity image of the whole group of cells. Light to dark colors indicate low to high total APL^*^ fluorescence intensity pixels and high to low fractional contribution of the AF to the total fluorescence intensity, with similar fractional contributions to the total pixel intensity. Phasor images at time zero show a dark gray color, characteristic of the phasor corresponding to the autofluorescence *AF*. (**d**) Corresponding phasor diagram associated to previous FLIM-phasor images, showing the black lines corresponding to linear combinations of 100% pure APL^*^-A and autofluorescence (A-AF_1_ for CH1; A-AF_2_ for CH2), and 100% pure APL^*^-B and autofluorescence (B-AF_1_ for CH1; B-AF_2_ for CH2). λ_exc_ = 750 nm. 1.2 ms/pixel.

Next, we have chosen [APL^*^] 5 nM labelling of HeLa-wt cells overtime as an example to summarize the whole set of spectroscopic images acquired in this study, for each cell and APL treatment condition. Figure 3a shows two-photon steady-state fluorescence intensity *XY* sections, corrected by G, determined simultaneously at CH1 (first row, *I*_*F1*_), and CH2 (second row, *I*_*F2*_). Figure 3b shows the Generalized Polarization *GP* images, (first row) and corresponding standard error *σ*_*GP*_ images (second row), determined from *I*_*F1*_ and *I*_*F2*_*, XY* fluorescence sections of the same group of HeLa-wt cells. Figure 3c shows the corresponding FLIM-phasor images. Figure 3d shows the phasor plots associated to the previous FLIM-phasor images. It is clearly observed that the formation of APL^*^-A species occurs most efficiently near the edges of the cells.

We have quantified the fractional contribution of APL^*^-A (*a*_*A*_), APL^*^-B (*a*_*B*_) species and AF (*a*_*AF*_) (see Methods) to the total intensities of each pixel measured at each emission channel, and then, we have re-normalized APL^*^-A and APL^*^-B fractional contributions for each emission channel, removing the corresponding AF contribution, (*α*_*A*_, *α*_*B*_, *α*_*A*_ + *α*_*B*_ = 1)_CH1_, (*α*_*A*_, *α*_*B*_, *α*_*A*_ + *α*_*B*_ = 1)_CH2_. Although for the moment, we can not translate these values into absolute concentrations of APL^*^-target species at each pixel, the variation of *α*_*B*_ determined at CH1 is chiefly sensitive to changes in APL^*^-B species populations. Analogously, the variation of *α*_*A*_ determined at CH2 would report mainly changes in APL^*^-A species populations. Note that in this study, (*α*_*B*_)_CH1_ + (*α*_*A*_)_CH2_ ≠ 1. It is important to note that (*α*_*A*_)_CH1_ and (*α*_*B*_)_CH2_ represent the non-negligible bleedthrough signals from APL^*^-A species at CH1, and APL^*^-B species at CH2. This fractional contribution is more important when the relative populations of A and B species are very different.

Supplementary Fig. S4a,b show the average fluorescence intensity changes per pixel at CH1 and CH2, respectively, corresponding to maximum APL labelling cell regions measured at each time, for HeLa-wt and HeLa-APL-R cells, treated with different [APL^*^]/[APL] concentrations. Supplementary Fig. S4c,d shows the re-normalized fractional contributions of APL^*^-B species (*α*_*B*_, referred to CH1), and APL^*^-A species (*α*_*A*_, referred to CH2), determined at each time for HeLa-wt and HeLa-APL-R cells. Supplementary Fig. S4d shows that about 50% of the total fluorescence signal per pixel at CH2, of HeLa-wt APL treated cells came from APL^*^-A complexes (*α*_*A*_ ≈ 0.5) at short interaction times, and remains nearly constant over time, except for [APL^*^] 2n M. Adding extra unlabeled APL, and/or washing with buffer/stirring the media did not result in any significant change in *α*_*A*_. At [APL^*^] of 2 nM and short times, *α*_*A*_ > 0.8, and later decreases to about *α*_*A*_ ≈ 0.5 as time goes by. At [APL^*^]/[APL] ≥100 nM, an increase in APL^*^-A relative populations was observed, reaching *α*_*A*_ values of about 0.7. For HeLa-APL-R cells, APL^*^-A species reached the 70–80% (*α*_*A*_ = 0.7–0.8) on CH2. HeLa-wt shows high *α*_*B*_ values since short times, except for [APL^*^] 2 nM, remaining nearly constant overtime (Supplementary Fig. S4c). Adding extra unlabeled APL, and or washing with buffer/stirring the media did not result in any significant change in *α*_*B*_. At [APL^*^] 2 nM, and short times APL^*^-B species signal represented less than 40% of the total fluorescence signal at CH1, *I*_*F1*_, (*α*_*B*_ ≈ 0.4), reaching about the 80% at long times (*α*_*B*_ ≈ 0.8). At [APL^*^]/[APL] ≥100 nM, a decrease in the fractional intensity of APL^*^-B species was observed over time, increases again after washing/stirring the media. In contrast, *α*_*B*_ was significantly reduced to 0.3–0.55 in HeLa- APL-R cells.

**Figure 4:**
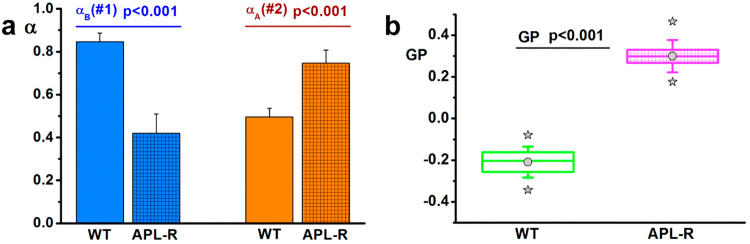
Changes in APL^*^-A and APL^*^-B relative populations and GP values at the “cytoplasm” of HeLa-wt and HeLa-APL-R cells. (**a**) Statistical comparison of the re-normalized fractional contributions of APL^*^-B (*α*_*B*_) to the total intensity measured at CH1, and APL^*^-A (*α*_*A*_) to the total intensity measured at CH2, respectively, for HeLa-wt (n = 20) and HeLa-APL-R cells (n = 20) treated with [APL^*^] 5–10 nM, *t* = 20–30 minutes. (**b**) Statistical comparison of the distribution of mean GP values at the “cytoplasm” of HeLa-wt (n = 20) and HeLa-APL-R cells (n = 20) treated with [APL^*^] 5–10 nM, *t* = 20–30 minutes, using box whisker plots. The box indicates the interquartile range from 25% to 75%, and the whisker indicates the ±1σ values. The middle line of each box indicates 50% quartile; grey circle and stars indicate the average, and the minimum and maximum GP values, respectively, for HeLa-wt and HeLa-APL-R cells. We found the observed changes to be statistically significant.

We have quantified the changes in APL^*^-A and APL^*^-B relative populations at the “cytoplasm” of HeLa-wt and HeLa-APL-R cells, treated with [APL^*^] 5–10 nM, at *t* = 20–30 minutes by quantifying their re-normalized fractional contribution to the total fluorescence intensity (*α*_*A*_ and *α*_*B*_) measured on CH1 and CH2 (see Methods), for n = 20 cells of each type. Figure 4a shows the corresponding mean *α*_*A*_ and *α*_*B*_ values. The error bars show the standard deviation calculated over fractional contributions obtained from each individual analyzed cell. Comparing the two re-normalized fractional contributions for HeLa-wt and HeLa-APL-R cells, we found that the differences in both, *α*_*A*_ and *α*_*B*_, determined for HeLa-wt and HeLa-APL-R cells are markedly significant (p < 0.001), with *α*_*B*_ ≫ *α*_*A*_ in HeLa-wt and *α*_*A*_ ≫ *α*_*B*_ in HeLa-APL-R cells, suggesting that APL^*^-B species seem to be critical for the mechanism of action of plitidepsin, while APL^*^-A species seems to be more related with cell defense against APL entry into cells. In the same way, we have made the statistical comparison of the GP values at the “cytoplasm” of the same HeLa-wt and HeLa-APL-R cells, treated with [APL^*^] 5–10 nM, at *t* = 20–30 minutes (n = 20 cells of each type). We have determined a mean GP value (±1 standard deviation, SD) for the “cytoplasm” of each cell, from the Gaussian fitting of the corresponding GP histogram obtained from the GP image (see Methods). Comparing the mean GP values determined for the “cytoplasm” of HeLa-wt and HeLa-APL-R cells, we found that the observed differences are markedly significant (p < 0.001).

## Discussion

Imaging of HeLa-wt cells treated with [APL^*^] 2 nM show interesting details during the first steps of APL interaction in living cells. During the first minute, APL may disturb cellular adhesion to the surface of the coverslip, so that the first image of APL treated HeLa-wt cells at the surface of the coverslip was always blurred. Then, cells seem to react to APL entry, re-establishing their contacts with the surface, and “returning” to be in focus in the next *XY* image. Interestingly, this effect was significantly less marked in APL treated HeLa-APL-R cells. Moreover, our results seem to indicate that APL^*^-B complexes are first formed on the “membrane” region, and then, somehow, overtime, they are distributed throughout the “cytoplasm”, while APL^*^-A complexes seem to be distributed in the “membrane” and “cytoplasm” from the first minutes of APL^*^ interaction.

Considering the spectroscopic properties of DMAC^*^, APL^*^-A complexes, mainly detected at the blue emission region, contain APL^*^ with DMAC^*^ in a nonpolar environment, in a biocavity probably protected from the aqueous media. It may be trapped within a group of biomolecules. APL^*^ in APL^*^-B complexes, mainly detected at the green emission region, could be more exposed to the aqueous media, though there could be some steric constraints at its binding site that may hinder the internal rotation of DMAC.

APL^*^–eEF1A2-GFP FRET efficiencies of about 80% were determined for the acceptor side FLIM phasor analysis, assuming an average donor lifetime of 2.6 ns^4^ (very close to τ_B_ = 2.45 ns; α_B_ ≈ 0.8 at CH1, and α_A_ ≈ 0.5 at CH2) for [APL^*^] 5–10 nM. In light of the results on the two APL^*^ species characterized in this study, we have reanalyzed the previous FLIM-FRET data, taking into account that in each pixel we may have two possible FRET donor samples, APL^*^-A and APL^*^-B molecular species, with different fractional contributions, and different emission spectra and lifetime properties, τ_A_ = 3.9 ns and τ_B_ = 2.45 ns. The previous FRET result in terms of high FRET efficiency determined for the acceptor channel phasor plot stands, although the introduction of the variation of the donor lifetime results in a greater uncertainty (E ∼ 50–80%). Then, from previous FRET results, we can assume that APL^*^-A and APL^*^-B complexes contain at least one subunit of the elongation factor eEF1A2^4^, but they could be multi-protein complexes. Taking into account the cellular distribution and regulated populations of APL^*^-A complexes at the “membrane” region, they could be associated in some way to cellular cortex structures/components. The amount of APL^*^-A complexes at the “membrane” neither appears to change with time, nor with [APL^*^]. The number of these species seems to be quite conserved in HeLa-wt and HeLa-APL-R cells. However the formation of APL^*^-A and APL^*^-B complexes at the “cytoplasm” is more dynamic. It is clearly observed that the formation of both APL^*^ species occurs most efficiently near the edges of the cells. Over time, APL^*^-A complexes appear strategically distributed throughout the “cytoplasm”, showing a non-uniform dynamic distribution, reorganizing themselves whenever the medium was washed or stirred, which suggests effects related to cytoskeleton macro-structures. In the case of HeLa-wt cells, APL^*^-A species were widespread throughout the “cytoplasm”, whereas for HeLa-APL-R cells, APL^*^-A species were accumulated at the edges of the cells, and near the nucleus. From the intensity images, and the estimated fractional contributions for HeLa-wt cells treated with [APL^*^] 2–10 nM, *t* < 60 minutes, we can qualitatively conclude that the amount of APL^*^-A species at the “cytoplasm” is much lower than APL^*^-B species, and their distribution is non uniform. However, at longer treatment times, and for [APL^*^]/[APL] ≥ 100 nM, suddenly a sharp increase in APL^*^-A fractional intensities was observed, due to an increase in the number of species and/or compaction of macro-structures to which they may be bound.

In HeLa-wt cells the fractional intensities of APL^*^-B species increase over time, to finally reach a plateau. The rate of formation of APL^*^-B species increases with [APL^*^]. This behavior is conserved until added [APL^*^] 10 nM. The formation of APL^*^-B complexes seems critical for the mechanism of action of plitidepsin, because these species were strongly inhibited in HeLa- APL-R cells. However, APL^*^-A species seem to be more related with cell defense against APL entry into the cell. EGCG 100 μM pre-treatment of cells slowdown the entry of APL into HeLa-wt and HeLa-APL-R cells. Our results suggest that EGCG alters in some way the formation of APL^*^-A species at the “membrane”, induces changes in APL^*^-A cellular distribution, which, in some way, translates into a slowdown in the formation of APL^*^-B species.

In summary, the results presented in this study demonstrate the ability of the two proposed technical approaches to monitorize, in real time, the formation, and relative subcellular distribution of APL^*^-A and APL^*^-B complexes in live cells. The FLIM-phasor approach permits a better quantification of the APL^*^-A and APL^*^-B complexes at each pixel. Nevertheless, GP imaging, despite its simplicity, has proved to be very informative to characterize the dynamics of different APL^*^ species, in a qualitative way.

More work is needed to ascertain the precise function(s) of eEF1A2 that is/are impaired by plitidepsin to induce tumor cell death so efficiently. Our results will complement future research efforts concentrated on elucidating the mechanism of action of this potent antitumor agent of natural origin.

The information obtained with *GP* and FLIM-phasor imaging, together with FLIM-FRET phasor imaging may complement and facilitate the global interpretation of all the results obtained with other biochemical and cell biology methods to determine and characterize the mechanism of action of APL. Effects, such as drug total concentration, exposure time, cell type, activators/inhibitors of the mechanism of action, can be explored in a systematic and relatively simple way. Still, further work has to be done to characterize all the proteins involved in both sets of APL^*^-complexes, and to try to measure their absolute concentrations overtime, in all the different cellular locations, taking into account changes in their fluorescence quantum yields and the cell autofluorescence fractional intensity (mainly from NADH), which are not negligible for regions showing low concentration of APL^*^-complexes. This knowledge may open new avenues to decipher the mechanism of drug action, and it can be extended to other biomolecular interaction studies, in which the target is distributed over a wide range of cellular locations, concentrations, showing canonical and non-canonical functions, as is the case of the elongation factor eEF1A2.

## Materials and Methods

### General

#### Cell lines and cell culture

HeLa cervix adenocarcinoma cells (ATCC CCL-2; HeLa-wt) were obtained from ATCC (Manassas, VA, USA). The stably plitidepsin resistant HeLa cell subline (HeLa-APL-R) was generated at PharmaMar as previously described^19^. Cells were maintained in Dulbecco’s Modified Eagle’s Medium (DMEM, Sigma, St Louis, MO, USA) supplemented with 10% fetal bovine serum (FBS), 2 mM L-glutamine and 100 units/mL of penicillin-streptomycin at 37 °C, 95% humidity and 5% CO_2_. Fluorescence studies were run in Thermo Scientific^TM^ Nunc^TM^ Lab-Tek^TM^ II chambered coverglass (8 wells; ∼8000 cells/well; 500 μL/well) at 37 °C. Before measurements at the micro-spectrometer, cells were washed with Tyrode-glucose buffer (NaCl 145 mM, KCl 4 mM, MgCl_2_ 1 mM, CaCl_2_ 1.8 mM, HEPES-Na 10 mM, glucose 10 mM), pH 7.4, just before starting the measurements.

*Epigallocatechin gallate* (EGCG) was purchased from Sigma (St Louis, MO, USA). HeLa-wt and HeLa-APL-R cells were pre-treated with EGCG 100 μM for 30 minutes, and then loaded with different plitidepsin concentrations.

### Plitidepsin and fluorescent coumarinated Plitidepsin analogue

Plitidepsin (C_57_H_87_N_7_O_15_, MW: 1109.6, CAS No 137219-37-5, APL), and a fluorescent coumarinated plitidepsin analog (PM01203; APL^*^) were prepared by PharmaMar (Colmenar Viejo, Spain). Stock solutions (1 mg/ml in DMSO, which is about 0.90 mM) were prepared and stored at −20 °C.

HeLa-wt and HeLa-APL-R cells were treated with different APL^*^/APL concentrations (2^*^ nM, 2*/3.3 nM, 5^*^ nM, 10^*^ nM, 10^*^/90 nM, 10^*^/440 nM, using APL^*^ as a fluorescent tracer, and followed the emission of APL^*^ in real time (the final DMSO concentration was always ≤ 0.5% in the cell samples).

A characteristic that we have observed and that has caught our attention, is that during the first minute after the addition of APL^*^ and/or APL to HeLa-wt and HeLa-APL-R cells, images showed cells slightly out of focus. On the next image, nevertheless, cells were again properly attached to the plate surface, without significant changes in their contact area. This behavior was more evident at low APL concentrations, requiring special care when treated cells were washed with Tyrode-glucose buffer.

### Fluorescence Micro-spectroscopy

Two-photon, two-color spectroscopic images were acquired simultaneously with a MicroTime 200 system (PicoQuant, Germany) coupled with a MaiTai laser (λ_exc_ 750 nm; 80 MHz), an Olympus IX71 inverted microscope (60x; 1.2 NA), and two single-photon counting avalanche diodes (*τ* - SPAD, PicoQuant, Germany), using the Time-Tagged Time-Resolved (TTTR) detection mode at 37 °C. TTTR mode allows the recording of every individual fluorescence photon from each pixel, together with its timing, emission color (CH1, CH2), and/or polarization properties. Once the acquisition of the image is finished, all the detected photons per pixel can be used to build steady-state fluorescence intensity images, or to produce fluorescence decay lifetime images (FLIM), using the ps-temporal resolution of the system and SymphoTime software (PicoQuant, Germany). Since we do not know a priori the composition and the emission spectra of APL^*^ cellular species, we have optimized the emission regions, to separate the emission from two sets of APL^*^-target species, named here as APL^*^-A and APL^*^-B, in terms of the polarity of APL^*^ microenvironment and their excited-state lifetimes. The filters used in this study were all from Semrock (Germany): CH1 (green emission; FF01 520/35); CH2 (blue emission; FF02 435/40); and dichroic filter (FF458-Di02). Even though at CH1 we mostly select APL^*^-B (more polar) molecular species, and at CH2, APL^*^-A (less polar) molecular species (Supplementary Fig. S1), we may have a non-negligible signal bleedthrough from each species in the other channel. Nevertheless, total fluorescence intensities per pixel will generally be the sum of the fractional intensities from APL^*^-A and APL^*^-B, complexes, and the cell autofluorescence.

### Generalized Polarization GP images

The Generalized Polarization parameter, *GP*, is a simple approach to quantitatively analyze the difference between pairs of fluorescence intensities *I1*, *I2*, measured simultaneously at two emission regions (CH1 and CH2). The difference between both intensities is effectively normalized dividing by their sum, pixel by pixel, to give a unit-less value, defined by:

1 $$GP=\frac{I2-I1\times G}{I2+I1\times G}=\frac{{I}_{F2}-{I}_{F1}}{{I}_{F2}+{I}_{F1}}$$

The *G* factor was introduced to take into account the differential wavelength effect on the sensitivity of the two detectors. It was determined by using a 40 nM APL^*^ solution in DMSO, comparing the fluorescence intensities measured at the microscope, in both channels, *Q1*, *Q2*, at different (*X*, *Y*) locations, and *Z* = 10 μm from the coverslip top surface, and the fluorescence intensities estimated from the corrected emission spectrum of the same APL^*^ solution, selecting the same spectral regions defined by the emission filters at the microscope, *F1, F2*:

2 $$G=\frac{Q2}{Q1}\times \frac{F1}{F2}$$

For the spectral conditions used in this work, G = 0.6, and it showed <3% variation across the imaging areas. *GP* is independent of the total fluorescence intensity per pixel, (*I*_*F2*_ + *I*_*F1*_), making it a useful imaging tool to compare at a glance, in a single panel the effect of different APL^*^ treatment conditions on the relative intensity changes, *I*_*F1*_*/I*_*F2*_ at each pixel, reflects the relative amounts of APL^*^-A and APL^*^-B species. The whole range for *GP* is −1 to +1. In this work, negative values would correspond to a greater proportion of APL^*^-B species (green color gradient scale), and similarly, positive values would correspond to a greater proportion of APL^*^-A species (magenta/blue violet color gradient scale).

In general, GP is not uniform within a single cell. GP population distributions were obtained from the histograms of the GP values corresponding to specific regions of each cell, and fitted to one, two or three Gaussian functions by the non-linear fitting algorithm (Origin 8.5.1). We assigned a mean GP value to the cytoplasm of each cell, corresponding to the more abundant population of GPs. Box-whisker plot shows the interquartile range of mean GP values, from 25% to 75%, and the whisker indicates ±1 σ values. The middle line of each box indicates 50% quartile. Grey circles show the estimated average GP values for the “cytoplasm” of HeLa-wt and HeLa-APL-R cells, and grey stars show the minimum and maximum GP values.

### Fractional contributions of APL^*^-A and APL^*^-B species to the total intensity from the Phasor Approach

**Figure 5:**
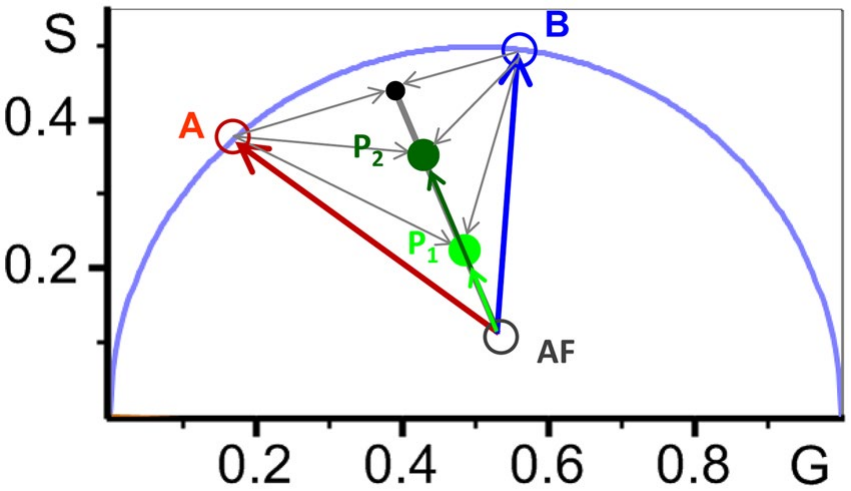
Quantification of fractional intensities of APL^*^-A complexes at CH2 and APL^*^-B complexes at CH1 from the FLIM phasor approach. Schematic illustration of the phasor plot corresponding to a single fluorophore in two different cellular microenvironments, (A and B), with single lifetimes *τ*_A_ (red cursor) and *τ*_B_ (blue cursor), *τ*_A_ > *τ*_B_. Point P1 corresponds to a mix of molecular species A and B, plus the cell autofluorescence AF. Point P2 would correspond to mix of molecular species A and B, with lower fractional contribution of AF. Re-normalized fractional contributions of A and B determined from P1 and P2, excluding the AF contribution, are the same, and they will match the values directly obtained from the black point at the intersection of the line joining A and B phasors (linear combination of A and B species), corresponding to a mixture of A and B species and 0% AF.

FLIM images were analyzed using the phasor approach^20–25^ for a three species system: two types of APL^*^ complexes, APL^*^-A (A phasor, *τ*_*A*_ = 3.9 ns) and APL^*^-B, (B phasor, *τ*_*B*_ = 2.45 ns), and the cell autofluorescence, AF, that changes with the wavelength emission range (see Fig. 5). AF phasor was determined for each cell type and detection channel, every day, before adding APL^*^ to the cells. The effect of the instrument response function was taken into account by recording FLIM images of a reference sample solution with known lifetime, and referencing all the phasors to this value. In this work we have used a solution of dimethyl-POPOP in ethanol (*τ* = 1.42 ns, measured under the same experimental conditions).

The blue arrow in Fig. 5, joining AF (pure AF) and B (pure APL^*^-B species) phasors marks one of the sides of the three species triangle in the phasor diagram. Along this line, every point (phasor) represents a different linear combination of 100% pure APL^*^-B species and AF, weighted by their fractional contribution (*α*) to the total pixel intensity. Analogously, the red arrow, joining the AF and A phasors, marks the other side of the triangle. Along this line, every point represents a different linear combination of 100% pure APL^*^-A species and AF. Point P2, inside the triangle, is a combination of the independent phasors of APL^*^-A, APL^*^-B, and AF, weighted by their fractional contributions to the total pixel intensity *a*_*A*_, *a*_*B*_*, a*_*AF*_ (Σ*a*_*i*_ = 1). Point P1 lies on the same line joining AF and P2, but closer to AF than P2. It represents a different combination of APL^*^-A, APL^*^-B, and AF, with a fractional contribution of AF to the total intensity of the pixel higher than that of P2 (*a*_*AF*_^*P1*^ > *a*_*AF*_^*P2*^). The re-normalized fractional contributions for APL^*^-A, APL^*^-B, after elimination of the AF contribution, are the same for P1 and P2 (*α*_*A*_^*P1*^ + *α*_*B*_^*P1*^ = 1; *α*_*A*_^*P2*^ + *α*_*B*_^*P2*^ = 1):

3 $${\alpha }_{A}^{P1}={\alpha }_{A}^{P2};{\alpha }_{A}^{P1}={(\frac{{a}_{A}}{{a}_{A}+{a}_{B}})}^{P1};{\alpha }_{A}^{P2}={(\frac{{a}_{A}}{{a}_{A}+{a}_{B}})}^{P2}$$

It is important to note that in general, to decompose a complex mixture into individual species using the phasor approach, the phasors corresponding to species A and B do not need to be located on the universal circle, *i.e*. they do not need to be single *τ* species.

### Data analysis

*GP* images were obtained using MATLAB^26^ software.

The FLIM-phasor data was analyzed using the SimFCS software developed at the Laboratory of Fluorescence Dynamics (LFD, UC Irvine). For statistical analysis of the fractional contributions (*α*), Student’s t-test was used to determine the significance and considered positive for p < 0.05. Sigma Plot software 13.0 was used.

## Electronic supplementary material

Supplementary Information
