# Supplementary material for: Dynamic cellular maps of molecular species: Application to drug-target interactions
Source: Sci Rep. 2018 Jan 18;8:1140. doi: 10.1038/s41598-018-19694-3 (PMC5773516; doi:10.1038/s41598-018-19694-3)
Supplement: Supplementary file 1 — Supplementary Information [file 41598_2018_19694_MOESM1_ESM.pdf]

Dynamic cellular maps of molecular species: Application to drug-target interactions

Carolina García[1], Alejandro Losada[2], Miguel A. Sacristán[1], Juan Fernando Martínez-Leal[2], Carlos M. Galmarini[2], M. Pilar Lillo[1*]

Supplementary information

Supplementary Note 1: Spectroscopic characterization of the fluorescent plitidepsin analog in solution

The spectroscopic properties of the fluorescent tracer, the coumarinated plitidepsin analog APL*, as well as the fluorophore DMAC*, would be determined by cumulative effects of polarity, viscosity, structural heterogeneity of the medium, and also specific solute-solvent and solute-protein interactions in the binding site. First we evaluated the spectroscopic properties of APL* in different solvents. We have measured the steady-state excitation and emission spectra, and the excited-state decays in polar protic (MeOH, EtOH, Tyrode-glucose buffer pH 7.4), polar aprotic (dimethyl sulfoxide, DMSO) solvents, and micelles (sodium dodecyl sulfate, SDS; octyl glucoside, OG). This is a summary of the results:

- The excitation spectra maximum wavelength (λ_{ex}=377 nm; Supplementary Fig. S1.a) essentially doesn't change with solvents. Only highlight the appearance of a shoulder in aqueous solutions, about 400 nm, indicative of the coexistence of at least two conformations/molecular species of APL* with DMAC* in different microenvironments.
- APL* solutions show broad emission spectra from 400 nm to 600-650 nm (Supplementary Fig. S1.a.), shifting to the red with increasing polarity of the solvent. The observed Stokes shifts for the different solvents were: 4200 cm^{-1}(DMSO), 4970 cm^{-1} (MeOH), 5430 cm^{-1} (2-15 mM SDS), 5380 cm^{-1} (34 mM OG), and 5560 cm^{-1} (H_2O, and Tyrode-glucose buffer, pH 7.4).
- In aqueous solutions, APL* shows two excited-state lifetimes (τ_1= 1.3 ± 0.1 ns; 80% and τ_2= 3.5 ± 0.2 ns; 20%), while the individual fluorophore DMAC* shows a single lifetime (τ= 1.3 ± 0.1 ns) (Supplementary Fig. S1.b.).
- In the presence of micelles of detergents SDS (15 mM) and OG (34 mM), the population of APL* species with excited state lifetime τ_2=3.5 ± 0.2 become majority (80-85%) (Supplementary Fig. S1.b.).
- In DMSO, EtOH, and MeOH, APL* presents a single lifetime of 3.5 ± 0.1 ns, 3.9 ± 0.1 ns, and 3.1 ± 0.1 ns respectively (Supplementary Fig. S1.b.)

The excited-state lifetime determined for diluted aqueous solutions of APL* (τ_1= 1.3 ± 0.1 ns), was similar to that measured for DMAC* alone, indicating that most of the APL* molecules, would present a conformation with DMAC* exposed to the aqueous media ($\lambda_{ex}\sim$ 400 nm). However, for all the other studied APL* solutions, included detergent micelles, regardless of polarity, most of the APL* species presented a long excited state lifetime in the range of 3.1-3.5 ns (80-100%). This result may indicate these solvents favor a more "closed" conformation of APL* (λ_{ex}=377 nm) with DMAC* interacting in some way with APL molecule, probably through strong intra- or inter-molecular hydrogen bond between the carbonyl group of the coumarin ring and a residue/residues of plitidepsin and/or steric

hindrances that prevent the free rotation of the dimethylamino substitution at the 7 position in DMAC*. According to these results, the excited state lifetime may be a sensor of changes in the characteristics of the microcavity/binding site of APL*, in the different APL*-target species in which it is involved.

Supplementary Figure S1:

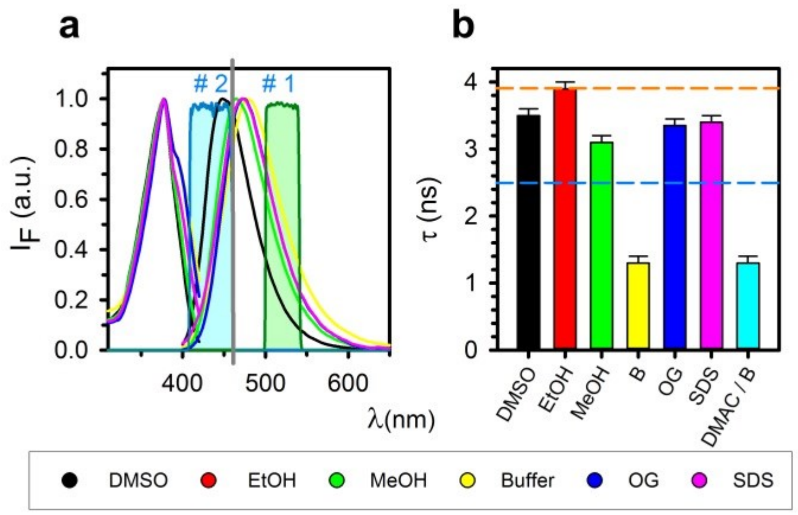

Supplementary Fig. S1. Fluorescence properties of APL* in solution. (**a**) Steady-state corrected and normalized excitation and emission spectra of APL* in different solvents: dimethyl sulfoxide (DMSO) (black), EtOH (red), MeOH (green), Tyrode-glucose buffer (B) pH 7.4 (yellow), octyl glucoside (OG) micelles (blue) and sodium dodecyl sulfate (SDS) micelles (pink). The transmission spectra of the emission band filters used in this work have been superimposed: FF02 435/40 (blue; channel *#2*) and FF01 520/35 (green; channel *#1*), and the dichroic filter FF458-Di02 (gray) (Semrock, Germany). (**b**) Excited-state lifetimes (τ) of APL* in different solvents, following the same color code as (a), comparing with DMAC* in Tyrode-glucose buffer pH 7.4 (light blue). The lifetimes determined for APL*-target complexes in living cells, APL*-A and APL*-B, are shown in dashed lines. λ_{exc}=375 nm; λ_{em}=475 nm.

Supplementary Figure S2:

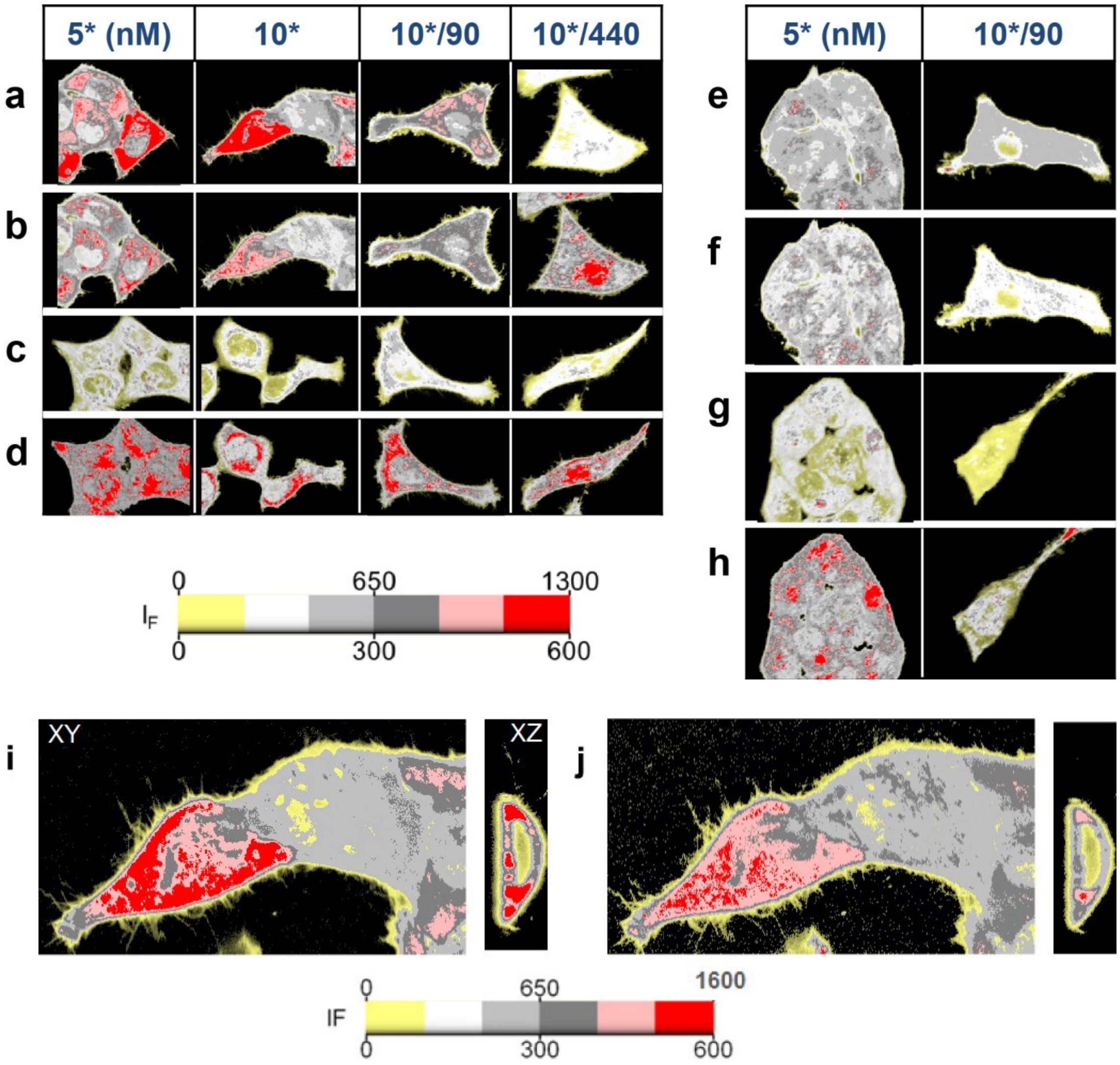

Supplementary Figure S2. Comparative study of APL* cellular labeling at the blue emission (CH1) and red emission (CH2) regions. Effect of APL concentration and EGCG 100 μM pre-treatment. Two –photon steady-state fluorescence intensity *XY* sections of representative groups of: (a,b) HeLa wt cells and (c,d) APL resistant HeLa APL-R cells; (e,-f) HeLa wt cells, and (g,h) HeLa APL-R cells pre-incubated with EGCG 100 μM for 30 minutes, treated with [APL*] 5* , 10* nM, and [APL*]/[APL] 10*/90 and 10*/440 nM, $t \sim 20$ minutes. (i,j) Enlarged *XY* fluorescence intensity sections ($t \sim 20$ minutes) and *XZ* ($t \sim 60$ minutes) of HeLa-wt cells treated with [APL*] 10 nM (same group of cells represented in a,b). CH1: (a,c,e,g,i) and CH2: (b,d,f,h,j).
Intensity values referred to a constant fluorescent tracer concentration of 10 nM. False color-code intensity scale yellow-red. λ_{exc}=750 nm. CH1: FF01 520/35 (maximum intensity scale IF: 1300 counts, a,c,e,g; and 1600 counts, i); CH2: FF02 435/40 (IF: 600 counts); Dichroic filter: FF458-Di02

Supplementary Figure S3:

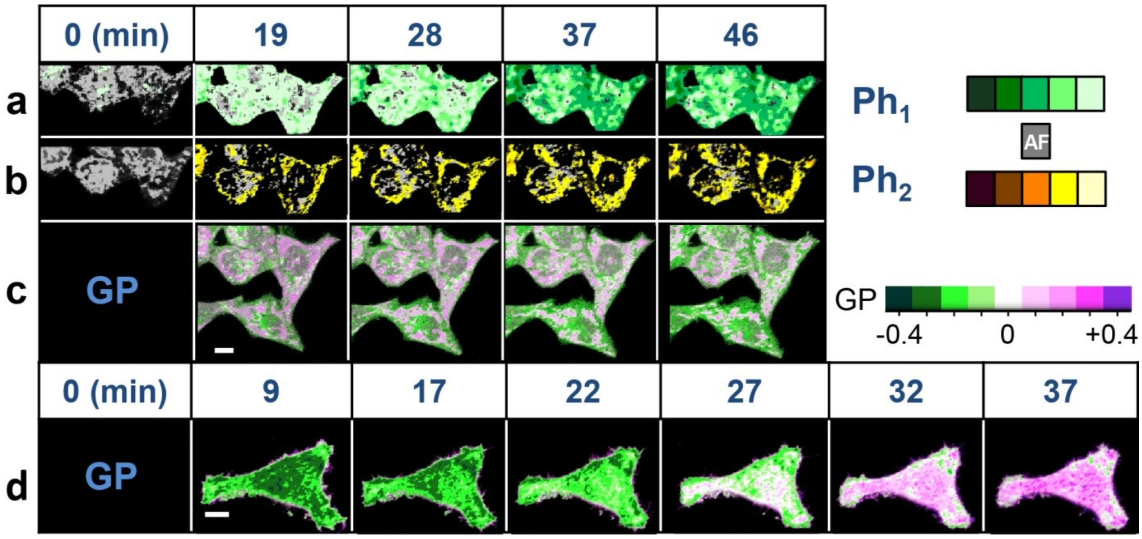

Supplementary Figure S3. HeLa wt cells treated with [APL*] 2 nM and [APL*]/[APL] 10*/90 nM

FLIM-phasor images of a representative group of HeLa wt cells treated with [APL*] 2nM at different times, showing regions in which coexist, with similar fractional contributions, APL*-A and APL*-B complexes, superimposed on a general grayscale intensity image of the whole group of cells. (a) (Ph_1; CH1; green scale). (b) (Ph_2; CH2; brown scale). Light to dark colors indicate high to low fractional contribution of the AF, indicative of the increase of the fractional intensities APL*-A and APL*-B species, maintaining their relative values. Phasor images at time zero show a dark grey color, characteristic of the *AF* phasor. The color pixels of FLIM-phasor images, Ph_1 and Ph_2, correspond to the clusters of APL*-target species identified in the phasor plot with the corresponding color cursors showed in Fig. 2 (main text). (c) Generalized polarization *GP* images of *XY* sections of the whole group of HeLa wt cells treated with [APL*] 2 nM (d) *GP* images of *XY* sections of a HeLa wt cell treated with [APL*]/[APL] 10*/90 nM at different times. Dark green-white-blue violet color *GP* scale: [-0.45,+0.45]. λ_{exc}=750 nm. CH1: FF01 520/35; CH2: FF02 435/40; Dichroic filter: FF458-Di02. 1.2 ms/pixel; 0.2 μm/pixel

Supplementary Figure S4:

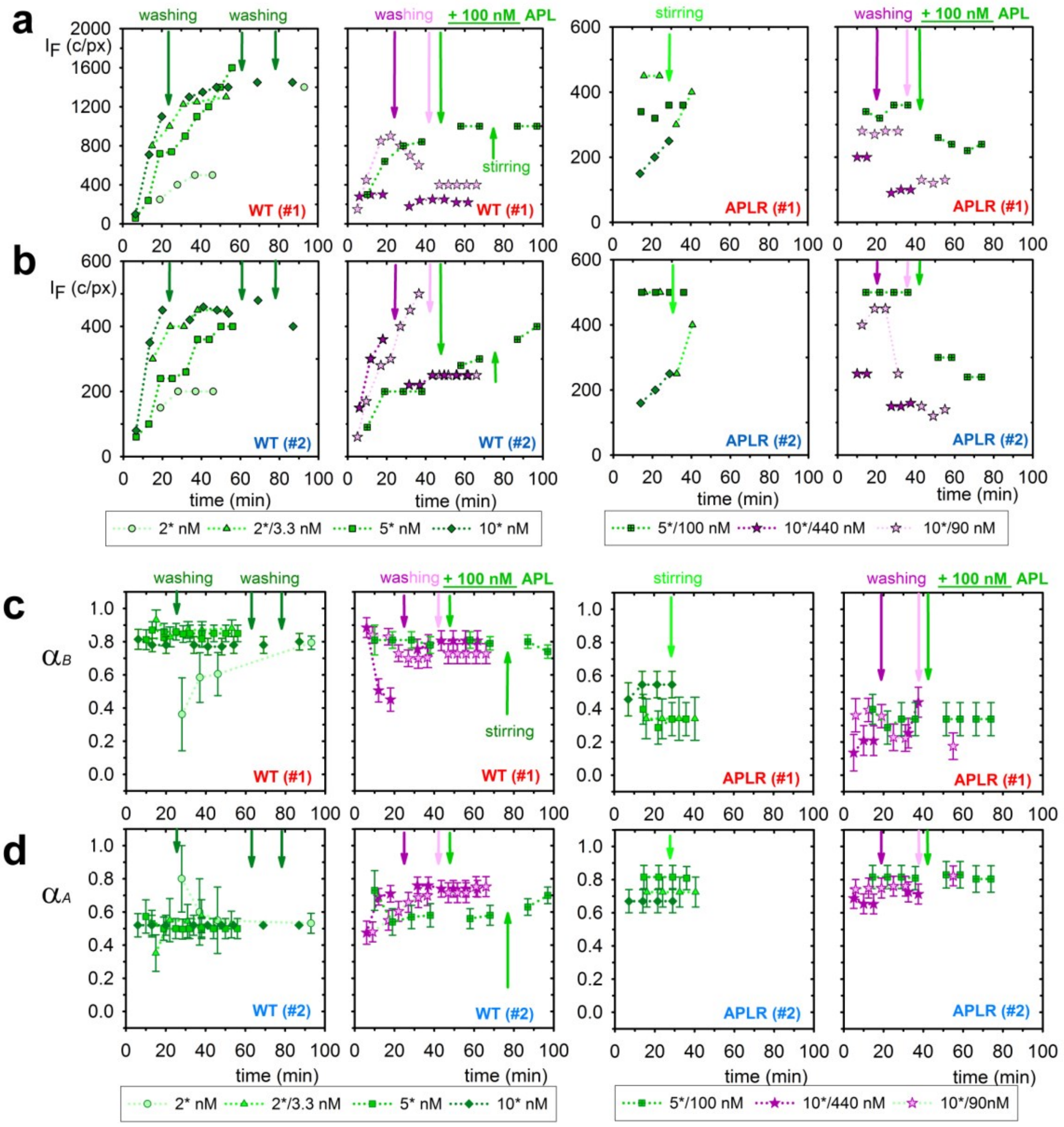

Supplementary Figure S4. Variation overtime of the average total fluorescence intensities *I_F (c/px)*, and re-normalized fractional contributions, α_A and α_B, to the total intensities of APL*-A and APL*-B species at the *cytoplasm* region. Fluorescence intensities, determined from the fluorescence images from (a) channel #1 (CH1), and (b) channel #2 (CH2), at the *cytoplasm* region of HeLa wt (WT) and APL resistant HeLa APL-R (APLR) cells, treated with [APL*] 2, 5 and 10 nM, and [APL*]/[APL] 2/3.3, 5/100, 10/90 and 10/440 nM. Effects of stirring and washing with fresh Tyrode-glucose buffer, and extra addition of 100 nM unlabeled APL. Intensities were estimated from *XY* sections of fluorescence images of treated HeLa wt and HeLa APL-R cells at the coverslip surface (*Z*=0). All intensity values are referred to 10 nM APL*. Re-normalized fractional contributions determined at the *cytoplasm* region from the phasor plot (see Methods) of (c) APL*-B (more polar) species (quantified from CH1 phasor plot), and (d) APL*-A (less polar) species (quantified from CH2 phasor plot). λ_{exc}=750 nm. Channel *#1*: FF01 520/35; Channel *#2*: FF02 435/40; Dichroic filter: FF458-Di02.

Supplementary Figure S5:

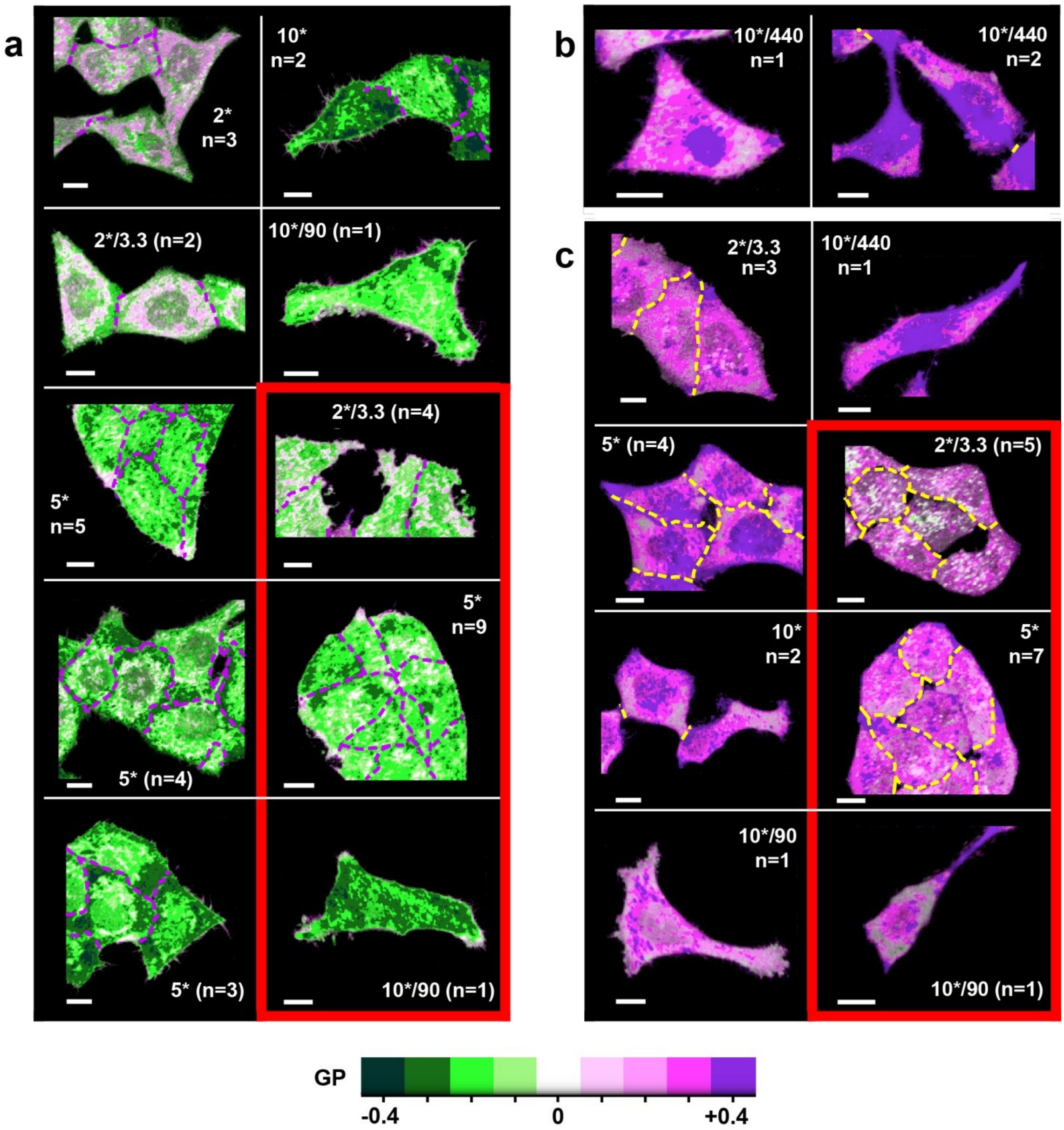

Supplementary Figure S5. Comparative panel showing a representative sample of analyzed HeLa-wt and HeLa-APL-R cells. Generalized polarization *GP* images of *XY* sections (at the surface of the coverslip; *Z*=0) of groups of (a, b) HeLa wt and (c) HeLa APL-R cells treated with different concentrations of added APL* (from 2* to 10* nM) and APL*/APL (10*/90 and 10*/440 nM) for $t \sim 20$ minutes. Red rectangle: HeLa wt and HeLa APL-R cells pre-incubated with EGCG 100 μM for 30 minutes. Scale bar 10 μm. Dashed lines separate different cells in each group. Dark green-white-blue violet color *GP* scale: Dark green and blue violet colors highlight cellular regions enriched in APL*-B (polar) and APL*-A (less polar) species, respectively. λ_{exc} =750 nm. CH1 FF01 520/35; CH2: FF02 435/40, Dichroic filter: FF458-Di02. 1.2 ms/pixel.
